# A review on targeted temperature management for cardiac arrest and traumatic brain injury

**DOI:** 10.3389/fnins.2024.1397300

**Published:** 2024-10-31

**Authors:** Hiroshi Ito, Sanae Hosomi, Takeshi Nishida, Youhei Nakamura, Jiro Iba, Hiroshi Ogura, Jun Oda

**Affiliations:** Department of Traumatology and Acute Critical Medicine, Osaka University Graduate School of Medicine, Suita, Japan

**Keywords:** body temperature, hypothermia, head injury, cardiac arrest, central nervous system

## Abstract

Therapeutic hypothermia inhibits organ damage by suppressing metabolism, which makes it a therapy of choice for treating various diseases. Specifically, it is often used to treat conditions involving central nervous system disorders where it is expected to positively impact functional prognosis. Although keeping the body temperature at a hypothermic level has been conventionally used, how to manage the body temperature correctly remains a topic of debate. Recently, the concept of temperature management has been proposed to improve the quality of body temperature control and avoid hyperthermia. This review focuses on the effect of temperature on the central nervous system in conditions involving central nervous system disorders and the practice of temperature management in clinical situations.

## 1 Introduction

Hypothermia management has a rich history, tracing back to ancient Egyptian writings from as far back as 5,000 years ago. In 1945, hypothermia was reportedly introduced into medicine through the use of cooling blankets (Dietrich and Bramlett, 2016; Fay, 1945). Hypothermia is believed to play an organ-protective role and is now used in a variety of fields, including organ transplantation and cardiac anesthesia. It has also been employed in the treatment of central nervous system (CNS) injuries such as head trauma and ischemic brain damage associated with cardiac arrest. Patients with head trauma and those who survive cardiac arrest are at high risk of neurological deficits that may impair their quality of life. Therefore, studies are being conducted to improve the functional prognosis in patients with CNS injuries. Temperature management therapy has received considerable attention in owing to its capability to offer substantial neuroprotection. To this end, the effects of targeted temperature management (TTM) have been studied in laboratory and preclinical studies in the clinical context of brain damage; however, its routine implementation faces several challenges. Indeed, despite of a dearth of uniform, evidence-based protocols for preventing fever after CNS injury, the optimal body temperature setting has been a subject of extensive debate. In recent years, the discourse has shifted towards the quality of temperature management, focusing on hyperthermia prevention, the rate of body temperature reduction, and the duration of temperature maintenance. However, the type of temperature management required for specific patient needs remains unclear. Therefore, in this review, we first discuss the pathophysiology of brain thermoregulation in the CNS. Next, we discuss the latest findings and future medical developments in the field of temperature management in head injuries and cardiac arrests, with a focus on the CNS.

## 2 Review

### 2.1 Relationship between increased body temperature and prognosis after brain damage

Fever occurs commonly following a head injury and can have a negative impact on the patient’s medical condition, affecting clinical outcomes. Approximately 73% of patients with head injuries develop a fever of 38°C or higher within the first week after injury, and the incidence and duration of fever are associated with the severity of injury, functional prognosis, and mortality (Stocchetti et al., 2002). The mortality rates in patients with head trauma with normal to low body temperature (<38°C), intermediate fever (38–39°C), and high fever (>39°C) have been reported to be 6.0, 13.6, and 37.0%, respectively, and the mortality rate increases with increasing body temperature (Li and Jiang, 2012). Similar to what is observed in patients with head trauma, there is an association between increased body temperature and functional prognosis in patients who become comatose following cardiac arrest (Zeiner et al., 2001). Thus, monitoring body temperature after brain damage is crucial in acute care. Furthermore, when body temperature rises, the brain temperature increases more than the body temperature (Rossi et al., 2001). Therefore, relying on body temperature alone may underestimate brain temperature (Thompson et al., 2003).

### 2.2 Pathophysiology of the brain damage and mechanisms of increased body temperature

**Figure 1:**
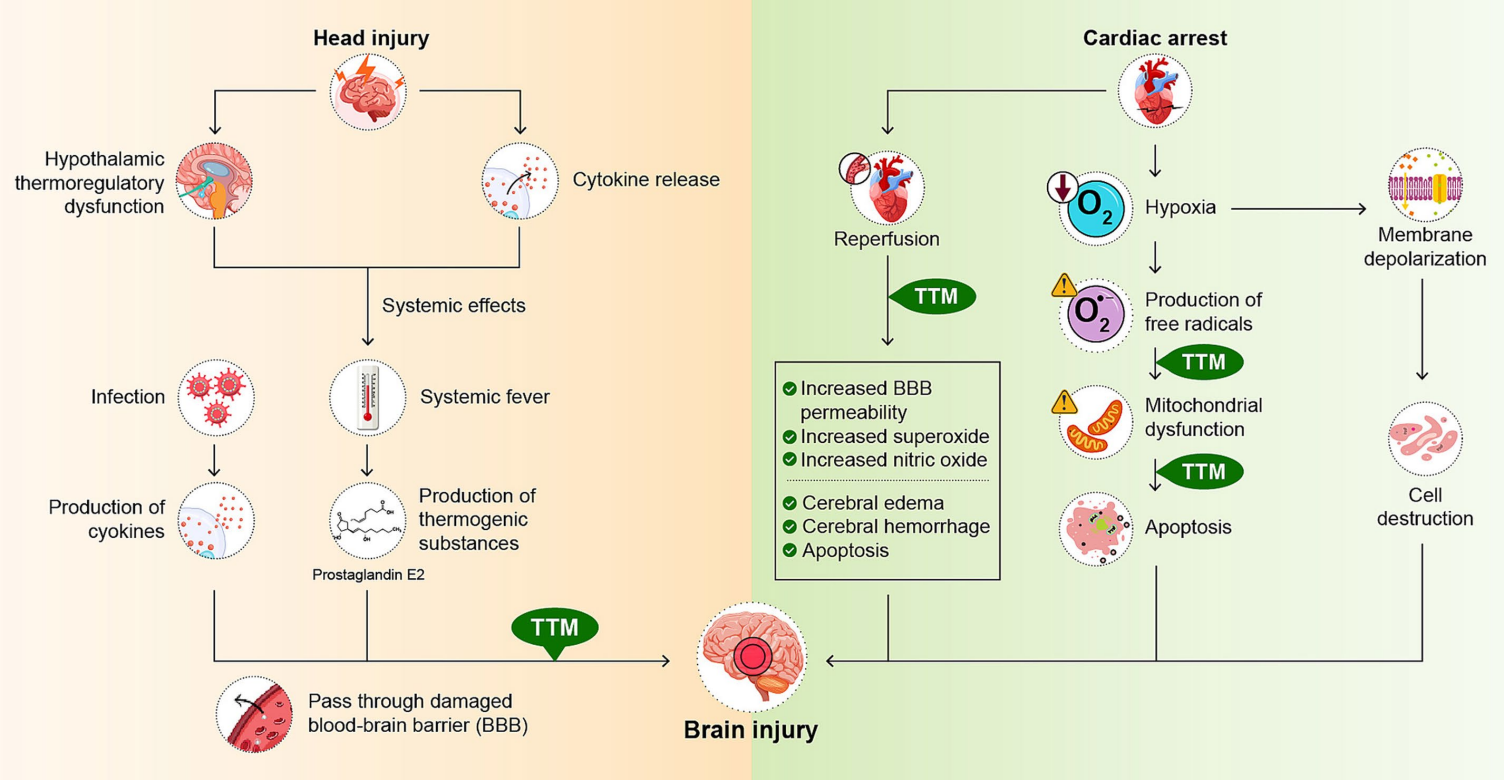
Mechanism of brain damage in head injury and cardiac arrest and effects of temperature management. Brain damage is a consequence of the head injury. This damage triggers the release of inflammatory cytokines. In addition, damage to the hypothalamus causes the body’s thermoregulatory system to fail. These factors have a systemic impact, resulting in fever. Systemic fever results in the production of thermogenic substances such as prostaglandin E2 (PGE2). Infectious complications can also cause fever and stimulate the production of inflammatory cytokines. These thermogenic substances and cytokines cross the damaged blood–brain barrier (BBB) and affect neurons. In cardiac arrest, cerebral blood flow is impaired and cell membrane depolarization occurs. This leads to cell swelling and destruction. As part of the inflammatory response, oxygen free radicals are also produced, resulting in mitochondrial dysfunction and further progression of neuronal necrosis and apoptosis. Reperfusion can further exacerbate central nervous system damage, leading to secondary superoxide surges, increased nitric oxide production, alterations in BBB permeability, cerebral edema, cerebral hemorrhage, and apoptosis. Temperature management is believed to reduce brain damage by mitigating mitochondrial dysfunction and apoptosis. TTM, targeted temperature management.

Numerous factors are involved in the mechanism of fever following a traumatic head injury. In the acute phase after head injury, inflammatory cytokines such as interleukin-1 (IL-1), IL-6, tumor necrosis factor-*α* (TNF-α), and interferon-*β* are released from the injured site following trauma (Thompson et al., 2003). The presence of these inflammatory mediators in the CNS triggers a febrile response. When systematic fever is observed, thermogenic substances are produced throughout the body. Prostaglandin E2 (PGE2), one such thermogenic substance, crosses the blood–brain barrier (BBB) that has been disrupted by the brain injury and affects neurons (Natale et al., 2000). Particularly in the hypothalamus, where thermoregulatory functions are located, PGE2 activates thermosensitive neurons, resulting in an increase in body temperature (Saper and Breder, 1994). In addition, fever can further increase cerebral vascular permeability, exposing cranial neurons to cytokines and further aggravating them (Dietrich et al., 1991). Similarly, endogenous cytokines (IL-1β, IL-6, and TNF-*α*) are released by monocytes and macrophages in the acute phase of fever owing to complications of infection during treatment. These cytokines activate the cyclooxygenase pathway, leading to PGE2 production and a febrile response in the CNS, which can cause secondary brain damage (Thompson et al., 2003) (Figure 1). If a patient develops a fever during treatment owing to infection or other causes, it is necessary to monitor for increased body temperature, since this may aggravate brain damage.

In cardiac arrest, cerebral blood flow (CBF) ceases, leading to anoxic cell membrane depolarization. This results in cellular expansion and destruction. Hypoxic brain neurons release glutamate, leading to increased intracellular calcium concentrations and further cellular damage. Ischemia also leads to the accumulation of lactate as well as glutamate, which, combined with mitochondrial dysfunction, can lead to metabolic acidosis. This acid toxicity is also known to induce neuronal cell death (Quillinan et al., 2016). In addition, oxygen free radicals are produced as part of a systemic and local inflammatory response, resulting in mitochondrial dysfunction and further progression of neuronal necrosis and apoptosis. Furthermore, restoration of CBF can also lead to the CNS damage, such as secondary superoxide surges, increased nitric oxide production, altered BBB permeability, cerebral edema, and apoptosis (Yokobori and Yokota, 2016; Galvin et al., 2015) (Figure 1). Accompanying such neuronal injury, damage to the hypothalamus, which is responsible for the thermoregulatory function are also thought to be factors in the development of fever (Zeiner et al., 2001).

### 2.3 Brain changes under high temperature conditions and countermeasures

Although whole-body hyperthermia has been used in various diseases, neurological complications such as cerebral edema, intracerebral hemorrhage, increased intracranial pressure (ICP), and demyelinating peripheral neuropathy have been reported. This is thought to be because the body’s ability to regulate brain pressure decreases when the body temperature exceeds 40°C (Cremer and Kalkman, 2007). Thus, the CNS is highly susceptible to the effects of heat. Additionally, in the acute phase of ischemic brain lesions owing to cardiac arrest or head trauma, the CNS is considered vulnerable and more susceptible to the effects of heat. Vascular permeability, edema formation, and inflammatory cell infiltration into the injured brain region are more exacerbated in situations of hyperthermia than under normal body temperature conditions. In patients with head trauma, impaired autoregulation of CBF results in changes in ICP, which also increases with increased body temperature. This is thought to be due to an increase in cerebral metabolism following the increased body temperature, which in turn increases CBF requirements (Nyholm et al., 2017) (Supplementary Table S1). Furthermore, when rats were placed in a hot environment after brain injury, mortality and total contusion were reported to increase, as well as long-term cognitive impairment (Dietrich and Bramlett, 2016; Sakurai et al., 2012; Dietrich et al., 1996; Baena et al., 1997; Titus et al., 2015). Strict control of body temperature is considered important in all patients with neurological injuries, since even an increase of only 1–2°C can result in neurological functional and histopathological deterioration in a hypoxic or ischemic brain (Cremer and Kalkman, 2007; Wass et al., 1995).

Therefore, neuroprotective therapies have been developed to prevent or reduce CNS damage caused by fever (Dietrich and Bramlett, 2016). Cooling improves cognitive function in a head trauma model with hyperthermia (Titus et al., 2015). Because hyperthermia significantly worsens the functional prognosis in head trauma and cardiac arrest, temperature management therapy is considered a clinically important therapeutic intervention to improve patient outcomes (Blaya et al., 2020).

### 2.4 Brain changes associated with lowering body temperature

In the brain damage associated with ischemia or trauma, even mild changes in brain temperature can have a significant impact on the brain. Lowering body temperature impacts calcium-dependent intercellular signaling controlling inflammation, brain edema, apoptosis, hemodynamics, and metabolism. Conversely, hypothermia reduces tumor necrosis factor receptor-1 (TNFR1) expression, which has been shown to have neuroprotective effects by affecting the regulation of apoptosis (Lotocki et al., 2006). Hypothermia also prevents apoptosis by inhibiting caspase-9 activation (Krech et al., 2017), decreasing glutamate (Boyko et al., 2013), and attenuating mitochondrial dysfunction (Ning et al., 2002). Indeed, lowering body temperature reduces the breakdown of the BBB and allows oligodendrocytes, which are responsible for myelination of neurons, to survive better (Jiang et al., 1992; Smith and Hall, 1996). Axons are protected, damage volume is reduced, and microglia responsible for immune function are less damaged and shift from inflammatory to anti-inflammatory (Atkins et al., 2017; Dietrich and Bramlett, 2010; Yokobori et al., 2013). Brain metabolism decreases by approximately 5–7% for each 1°C decrease in body temperature (Finkelstein and Alam, 2010). The decrease in metabolism causes a contraction of cerebral blood vessels and a decrease in ICP owing to a decrease in intracranial blood volume (Lee et al., 2010; Sandestig et al., 2014). In addition, hypothermia has been noted to have neuroprotective effects because the decrease in CBF leads to a decrease in cerebral edema, decrease in metabolism, decrease in acidity of reactive oxygen species, and suppression of apoptosis (Cai et al., 2023) (Supplementary Table S1). Selective brain cooling exerts systemic effects beyond the brain, significantly reducing intestinal barrier disruption, systemic inflammation, and peripheral vital organ damage (Chao et al., 2021). In animal studies, hypothermia after trauma has a beneficial effect on motor and cognitive function as well as histopathological changes (Clifton et al., 1991). In addition, hypothermia management has been associated not only with recovery of exercise and cognitive function, but also with reduced mortality and reduced weight loss (Clifton et al., 1991). Furthermore, a meta-analysis of preclinical cardiac arrest studies in animals reported that TTM confers significant neuroprotective benefits compared to normothermia, with the magnitude of improvement correlating inversely with the target temperature (Arrich et al., 2021).

### 2.5 Methods of temperature management in human patients

In the event of fever in patients with CNS injuries, such as head trauma or cardiac arrest, various methods have been incorporated to control fever. Acetaminophen, indomethacin, diclofenac, barbiturates, and propranolol have been used as antipyretics (Meythaler and Stinson, 1994; Cormio et al., 2000; Cairns and Andrews, 2002; Dippel et al., 2003). Physical cooling methods, including the use of fans, alcohol rubs, ice packs, and gastric lavage with cold saline, have also been implemented. However, the efficacy of these methods has been reported to vary, with both limitations and benefits observed. Some reports show that physical cooling alone lowers the mean core temperature by only 0.32°C, antipyretics alone by 0.58°C, and a combination of the two by only 0.54°C (Cremer and Kalkman, 2007; Stocchetti et al., 2002). Bernard et al. (2002) reported that hypothermia could be adequately managed using ice packs. Therefore, various devices have been developed in recent years to enhance the effectiveness of temperature management.

Nasal cooling is a method in which a mixture of perfluorocarbon and high-flow oxygen is blown intranasally through a catheter. This technique aims to cool the nasopharynx, which is expected to have a cooling effect deep in the brain via blood flow in the internal carotid artery, anatomically located close to the nasopharynx. Additionally, it dissipates heat at the base of the skull (Hong et al., 2022). In the PRINCE trial, a multicenter randomized trial using this nasal cooling, patients who underwent cardiopulmonary resuscitation (CPR) within 10 min after cardiac arrest were associated with improved survival to hospital discharge and neurological outcome compared to standard care (Castrén et al., 2010). However, this technique required a longer time to reach the target temperature after cooling was initiated, suggesting that it might be more effective in combination with other strategies such as intravenous cooling infusion or surface brain cooling (Hong et al., 2022).

Another means of selectively reducing head and neck temperatures has been developed: surface cooling. Specifically, surface cooling with a cap or neckband has been used in patients. The use of cooling helmets in patients with severe head trauma with a Glasgow Coma Scale (GCS) score of 8 or less did not improve mortality (Harris et al., 2009). In contrast, when cooling was combined with a cooling cap and neck band, selective cooling of the head and neck area was achieved while maintaining body temperature within the normal thermal range. The mean ICP was significantly lower compared to the usual treatment group, and the good functional prognosis at 6 months was also higher (68.9 vs. 46.7%, respectively, *p* < 0.05) (Qiu et al., 2006). Although no severe complications have been reported with this means of surface cooling, whether the temperature in the deep brain is sufficiently reduced has not yet been evaluated, and further studies are needed (Hong et al., 2022; Assis et al., 2019).

Another invasive cooling method involves the use of an intravascular cooling device, in which a catheter irrigated with cooling water in a closed circuit is placed intravenously (Thompson et al., 2003). In a phase I study, Marion (2001) compared the use of conventional cooling versus endovascular cooling (Alsius COOLGARD) in patients with head injuries; they found that fever in patients using the endovascular cooling device reduced in less than half the time compared to the control group, with no significant complications. It has also been reported that the device enabled patients to reach their target body temperature in a median of 3 h and is expected to be effective in lowering ICP in patients with poor ICP control (Sahuquillo et al., 2009).

In addition to these strategies, various cooling methods have been reported, such as the use of cooling pads on the body surface and the direct administration of a cooling infusion to the internal carotid artery. Each cooling method has its own set of complications, necessitating a thorough consideration of the risks and benefits associated with each method (Assis et al., 2019; Christian et al., 2008). Three trials compared endovascular cooling using surface cooling in cardiac arrest. These trials found no difference in survival to discharge or 28 days or in neurologic outcomes (Pittl et al., 2013; Deye et al., 2015; Look et al., 2018). The European Resuscitation Council and the European Society of Intensive Care Medicine guidelines for cardiac arrest state that both techniques should be recommended when cooling is necessary, although the evidence is less certain (Sandroni et al., 2022). Another guideline states that endovascular cooling may be better managed (Wyckoff et al., 2022). Although endovascular cooling systems are not explicitly recommended in the guidelines, it is suggested that their use may be an option in the future.

Although various cooling methods have been developed, it is crucial to consider not only the cooling method but also the duration of cooling and the rate of rewarming. Animal experiments have shown that rapid warming from hypothermia further aggravated axonal damage caused by trauma. In contrast, slow warming has been found to protect damaged axons (Suehiro and Povlishock, 2001). In animal models of ischemic brain damage, rapid warming after hypothermic control also resulted in higher expression of cytokines such as IL-1β and TNF-*α* and a worse functional prognosis compared to normal temperature control (Zhu et al., 2016). Given that patients with head trauma or cardiac arrest may be managed by lowering body temperature, the rate of temperature recovery after cooling should be carefully considered (Thompson et al., 2003).

Therefore, the concept of quality of temperature management has been discussed in recent years. When the target temperature for temperature control was changed from 33°C to 36°C at a single institution, compliance with temperature control deteriorated and frequency of fever increased. The quality of temperature management therapy was evaluated using following three parameters: time to goal, temperature fluctuation, and fever incidence; the quality of temperature management therapy improved with learning and training in a strict temperature management protocol (Strålin et al., 2022). Even with an established temperature management protocol, accurate management of body temperature presents a new challenge.

Temperature management requires consideration of the timing of induction, cooling rate, cooling method, maintenance of target body temperature, and rate of temperature restoration (Figure 1). In 2009, the concept of TTM was introduced as a replacement of the previous term “therapeutic hypothermia” with the consensus of five academic societies, emphasizing the quality of temperature management of the patient’s body temperature (Nunnally et al., 2011). Rigorous, scientifically based procedures are said to be important. However, there is still a lack of sufficient clinical data and knowledge regarding these optimal methods (Taccone et al., 2020).

### 2.6 Clinical studies of temperature management

Randomized controlled trials (RCTs) have reported that TTM for head trauma was not effective in improving functional outcomes (Cooper et al., 2018; Andrews et al., 2015). However, there are various potential biases and lack of consistent design in these reports; these studies included severe cases, such as patients with a GCS of 8 points or less and ICP of 20 mmHg or more, and it is possible that severity of illness may have affected the effectiveness of hypothermia therapy. The study by Andrews et al. also included patients in whom up to 10 days had elapsed since the injury. Furthermore, the time since injury and other therapeutic interventions may have had an impact, because there was no detailed description of therapeutic interventions other than hypothermia, such as hyperventilation or the use of hypertonic saline solution. At the same time, other reports mention the benefit of temperature management. A systematic review on temperature control therapy for head injury was limited to studies addressing the role of hypothermia in patients undergoing surgery after traumatic brain injury (Galvin et al., 2015) and those evaluating the benefits of maintaining a temperature of 35–37°C (Lewis et al., 2020). While some of these studies have examined the effects of different temperature controls, others examined the quality and timing of cooling. A study evaluating the duration of temperature control compared the effects of long-term (5 days) and short-term (2 days) mild hypothermia in patients with severe adult head trauma and found that the outcomes were improved with mild hypothermia when cooling was maintained longer than 48 h (Jiang et al., 2000). More recently, an RCT found that long-term mild hypothermia (34–35°C for 5 days) in severe head trauma was effective in improving outcomes in patients with high ICP (>30 mmHg) (Hui et al., 2021). In addition, in patients with head injury with an abbreviated injury scale (AIS) score of 3–4 temperature management to avoid fever significantly reduced mortality compared to mild hypothermia (Hifumi et al., 2016). Some reviews suggest that management to control fever improves outcomes in patients with head trauma, and others believe that avoidance of fever has a beneficial effect (Madden and DeVon, 2015; Chesnut et al., 2020). Moreover, some reports have examined the rate of rewarming. Slow warming over 48 h in patients with head trauma following hematoma removal improved long-term neurologic outcomes (Kaneko et al., 2018). A meta-analysis using a cooling index to evaluate the degree of cooling found that a high cooling index indicates adequate cooling and a benefit of hypothermia (Olah et al., 2018). A sub-analysis of the results of the POLAR study using this cooling index reported similar results (Olah et al., 2021). Further validation of these differences in temperature management protocols will be needed.

Bernard et al. (2002) and the Hypothermia After Cardiac Arrest (HACA) study group (2002) laid the groundwork for temperature control in cardiac arrest, comparing a group of cardiac arrest patients maintained at 33°C for 12 h with a normothermia group. The HACA group conducted a comparative analysis between normothermia and mild therapeutic hypothermia, maintaining the core body temperature between 32°C and 34°C for 24 h, in patients resuscitated from cardiac arrest due to ventricular fibrillation. Both concluded that hypothermic management strategies increased the proportion of patients with a favorable outcome. A comparative analysis of these two studies reveals notable disparities in their methodologies, encompassing differences in patient selection criteria, target temperature ranges, and durations of temperature management protocols. In other words, temperature control therapy for cardiac arrest patients’ needs to consider patient characteristics and variations in therapeutic interventions. This suggests that determining the optimal temperature management for prognosis in specific patients is highly complex. Since these initial studies, various comparative studies of target patients and temperature management have been conducted.

Studies comparing eligible patients have reported that the time to return of spontaneous circulation (ROSC) is related to functional prognosis (Sawyer et al., 2020) and that the severity of illness is differentially associated with temperature management therapy (Callaway et al., 2008).

There are also various reports of sex differences in patient outcomes for out-of-hospital cardiac arrest (OHCA). For example, one study found that a higher percentage of women who underwent extracorporeal cardiopulmonary resuscitation (ECPR) had better neurological prognoses (Springer et al., 2024). Another study reported that, after adjusting for in-hospital treatment, women had better functional outcomes at discharge (Lee et al., 2024). Conversely, several systematic reviews report no difference in 30-day adjusted survival after resuscitation between men and women (Hoedemaekers et al., 2022; Laura et al., 2023). Thus, while numerous studies have focused on sex comparisons, there have been few reports on the relationship between sex and temperature control. Among these studies, Park et al. (2023) reported no difference in neurological outcomes at 6 months between men and women in OHCA patients treated with TTM.

In comparing temperature management strategies, Nielsen et al. (2013), Lascarrou et al. (2019), and Dankiewicz et al. (2021) have conducted studies examining hypothermic versus normothermic temperature management, or specific temperature settings. A meta-analysis by Taccone et al. (2024) concluded that hypothermia at 33°C did not improve survival or functional outcomes in cardiac arrest patients with non-shockable rhythms. Various guidelines have started to emphasize body temperature control. In 2023, the American heart association (AHA) updated its recommendation to maintain a constant temperature between 32°C and 37.5°C. However, evidence supporting temperature management therapy in different subgroups of cardiac arrest patients remains inadequate (Perman et al., 2024).

In contrast, the 2022 ILCOR consensus recommends a target temperature of 37.5°C or lower to prevent fever, and it is unclear whether it is more beneficial to target a hypothermia of 32–34°C (Wyckoff et al., 2022). In other words, different strategies are recommended for temperature control in the first 24 h after cardiac arrest, namely, sustaining temperature control and preventing fever. The duration and methods of continuing temperature control and the differences in rate of rewarming need to be considered; however, there is scarcity of data on these topics. A study on the duration of temperature control showed no difference in outcomes between durations of 24 and 48 h (Kirkegaard et al., 2017). A clinical trial (NCT04217551) is presently underway to determine if increasing durations of induced hypothermia (6 to 72 h) affect neurological outcomes and to identify the optimal duration of induced hypothermia for neuroprotection in comatose survivors of cardiac arrest. Studies comparing means of temperature control showed no difference between surface cooling and the use of intravascular cooling (Pittl et al., 2013; Deye et al., 2015; Look et al., 2018). Only few studies have evaluated the prognostic impact of the time spent to rewarming. An examination of the effect of 12 and 48 h of rewarming on functional prognosis, revealed that there was no difference between the two durations (Hassager et al., 2023) (Table 1). Although it would be better to aim for gradual warming, the specific rate of warming has not been evaluated in detail. Future studies and meta-analysis are expected to elucidate and delineate the effects of different temperature management techniques.

**Table 1:** Major clinical trials comparing temperature control therapy.

Disease	Reference	Subject	Comparison	Duration of temperature management	Rewarming speed	Functional outcome	Mortality or complications
Traumatic brain injury	Clifton et al. (2001)	GCS: 3–8 with non-penetrating head injury	Hypothermia (33°C) vs. normothermia (37°C)	48 h	0.5°C/2 h	57% vs. 57% (RR 1.0; *p* = 0.79) for GOS:1 ~ 3 at 6 months	Hypotension: 10% vs. 3% (*p* = 0.01)
	Clifton et al. (2011)	TBI patients (aged 16–45 years, non-penetrating brain injury, and not responsive to instructions)	Hypothermia (33°C) vs. normothermia (37°C)	48 h	0.5°C/2 h	60% vs. 56% (RR 1.08; *p* = 0.67) for GOS:1–3 at 6 months	No difference in mortality at 6 months (*p* = 0.52)
	Maekawa et al. (2015)	Severe TBI patients (GCS: 4–8)	Hypothermia (32–34°C) vs. fever control (35.5–37°C)	72 h	≤1°C/day	53% vs. 48% (RR 1.24; *p* = 0.597), poor prognosis at 6 months	No difference in mortality at 6 months (*p* = 0.18)
	Andrews et al. (2015)	TBI patients who are sedated and on a ventilator, with an ICP >20 mmHg	Hypothermia (32–35°C) vs. standard therapy	48 h	0.25°C/h	OR 1.53 (*p* = 0.04) for GOS-E1–3 (poor prognosis) after 6 months	
	Hifumi et al. (2016)	AIS; 3–5 head trauma patients	Fever management group (35.5–37°C) vs. hypothermia (32–34°C)	72 h		No difference in GOS at 6 months (64.5 vs. 51.1%, *p* = 0.26)	TBI-related mortality better in fever management group 9.7 vs. 34.0%, *p* = 0.02
	Cooper et al. (2018)	Severe head injury	Hypothermia (33–35°C) vs. normal body temperature 37°C	72 h	0.25°C/ h	No difference in GOS-Ex; 5–8 after 6 months (48.8 vs. 49.1%, *p* = 0.94)	Pneumonia 55.0 vs. 51.3% (*p* = 0.4), increased intracranial bleeding 18.1 vs. 15.4% (p = 0.7)
	Hui et al. (2021)	GCS;4–8, ICP > 25 mmHg head injury patients	34–35°C for 5 days vs. 37°C control	5 days	0.25°C/4 h	OR for GOS (1–3 poor) after 6 months; 1.55, *p* = 0.105,	Mortality *p* = 0.111 after 6 months, difference in better prognosis only for ICP > 30 mmHg (60.82% vs. 42.71%; OR, 1.861; *p* = 0.039)
Cardiac arrest	Bernard et al. (2002)	Post-ROSC patients with coma, and initial cardiac rhythm of ventricular fibrillation	Hypothermia (33°C) vs. normothermia	12 h		49% vs. 26%, good outcome (*p* = 0.046)	Mortality at 30 days (51% vs. 68%; *p* = 0.145)
	HACA (2002)	Post-ROSC patients with ventricular fibrillation	Hypothermia (32–34°C) vs. normothermia	24 h	8 h to return to normal body temperature	55% vs. 39% (RR:1.40); CPC:1 or 2 with good functional prognosis at 6 months	Mortality at 6 months (41% vs. 55%; RR, 0.74)
	Castrén et al. (2010)	Post-ROSC patients with witnessed out-of-hospital cardiac arrest	Nasal cooling vs. standard therapy			CPC with good functional prognosis at discharge; 1 or 2 no difference (34% vs. 21%, *p* = 0.21)	Survival discharge (44% vs. 31%; *P* = 0.26)
	Pittl et al. (2013)	Post-ROSC patients with IHCA and OHCA	Invasive (Cool-Gard) vs. noninvasive (ArcticSun)	24 h	0.2–0.3°C/h	CPC 1 or 2; no difference (14% vs. 14%, *p* = 0.99)	Bleeding complications more common in invasive group (43.6 vs. 17.9%; *p* = 0.03)
	Nielsen et al. (2013)	GCS < 8 on admission to the hospital after OHCA	Hypothermia (33°C) vs. normothermia (36°C)	36 h	0.5°C/h	No difference in poor prognosis at 180 days (54% vs. 52%; RR, 1.02; *p* = 0.78)	No difference in mortality at 180 days (50% vs. 48%; RR 1.06; *p* = 0.51)
	Deye et al. (2015)	Post-ROSC patients with OHCA considered cardiogenic	Intravascular cooling vs. body surface cooling			No difference in CPC:1 or 2 after 28 days, (OR 1.41, *p* = 0.107)	Time to reach 33°C was shorter with intravascular cooling (*p* < 0.001). Complications were more observed (*p* = 0.009).
	Kirkegaard et al. (2017)	Post-ROSC patients with OHCA considered cardiogenic	33°C, 48 h vs. 24 h	48 h or 24 h	0.5°C/h	No improvement in CPC:1 or 2 at 6 months (69% vs. 64%; RR:1.08; *p* = 0.33)	Adverse events were more common in the 48 h group than in the 24 h group (48 vs. 24; 97% vs. 91%; *p* = 0.03)
	Lascarrou et al. (2019)	Post-ROSC patients with OHCA or IHCA with non-shockable rhythms	Moderate hypothermia (33°C) v.s targeted normothermia (37°C)	24 h	0.25–0.5°C/h	10.2% vs. 5.7% in CPC:1 or 2 at 90 days (P = 0.04)	No difference in mortality at 90 days (81.3% vs. 82.2%)
	Nordberg et al. (2019)	OHCA patients with bystander-witness	Prehospital trans-nasal cooling vs. standard cooling care after hospital arrival	24 h	0.2–0.5°C/h	16.6% vs. 13.5% in CPC:1 or 2 at 90 days (RR 1.23, *p* = 0.25)	No difference in mortality at 90 days (17.8% vs. 15.6%; *p* = 0.44)
	Dankiewicz et al. (2021)	OHCA patients who were comatose	Hypothermia (33°C) vs. normothermia with early treatment of fever (≥37.8°C)	24 h	0.25–0.5°C/h	No difference in functional outcomes (≥mRS ≥ 4) at 6 months (55% vs. 55%)	No difference in mortality at 6 months (50% vs. 48%; RR 1.04; *p* = 0.37)
	Hassager et al. (2023)	Post-ROSC patients with OHCA considered cardiogenic	12 h vs. 48 h until 37°C rewarming of temperature	24 h	1°C/12 h or 48 h	No difference in mortality or CPC:3 or 4 within 90 days (32.3% vs. 33.6%; *p* = 0.7)	No difference in mortality at 90 days (32.3% vs. 33.6%; *p* = 0.70)

RCT, randomized controlled trial; GCS, Glasgow coma scale; RR, risk ratio; GOS, Glasgow outcome scale; TBI, traumatic brain injury; ICP, intra cranial pressure; OR, odds ratio; GOS-Ex, Glasgow outcome scale extended; AIS, abbreviated injury scale; ROSC, return of spontaneous circulation; CPC, cerebral performance category; IHCA, in-hospital cardiac arrest; OHCA, out-of-hospital cardiac arrest; mRS, modified Rankin scale.

### 2.7 Hypothermia and complications

While hypothermia is expected to improve the prognosis of CNS function, various systemic complications may occur during hypothermic management.

Hypothermia in patients with severe head injuries suppresses Heat Shock Protein (HSP) 60 expression in polymorphonuclear cells, which induces innate immunity by activating dendritic cells and other cells (Quintana and Cohen, 2011). Specifically, hypothermia may suppress innate immunity and increase the risk of infection (Hashiguchi et al., 2003). The TTM2 study reported that inflammatory responses were not altered by hypothermia (Bro-Jeppesen et al., 2014) and hypothermia did not increase the risk of infection complications (Dankiewicz et al., 2017). In a per-protocol analysis of hypothermia management for traumatic brain injury by Andrews et al. (2015) complications of pneumonia were increased in the hypothermia group (70.5% in the hypothermia group) and 57.1% in normothermia group; absolute risk difference, 13.3% (95% confidence interval, 2.4 to 24.2%; *p* = 0.02). Several clinical trials have reported prophylactic antimicrobials for cardiac arrest and severe traumatic brain injury as a strategy against pneumonia (Francois et al., 2019; Fizelier et al., 2024). In the future, prophylactic antimicrobials for pneumonia during TTM management may be a strategy. Protocolized care may be needed to effectively monitor infections, as detecting fever associated with infection can be challenging under these conditions. Changes in the cooling system of the body may also be a sign of infection, as the workload of the cooling system increases rapidly when infection progresses and is accompanied by heat production. Hypothermic management increases the risk of bleeding by inducing thrombocytopenia, platelet dysfunction, and coagulopathy (Polderman, 2009; Rohrer and Natale, 1992). In the Andrew et al. study, hypothermia was discontinued early in 19% of cases at the clinician’s decision. There may be a trend toward avoiding the introduction of hypothermia in patients at high risk for rebleeding. Some studies have found an increase in cerebral hemorrhage with hypothermia management, while other reviews found no change in the frequency regarding cerebral hemorrhage (Cooper et al., 2018; Chen et al., 2019). Strategies for coagulopathy include transfusion therapy, ICP monitoring, CT follow-up, and frequent blood tests to assess for coagulopathy. Because traumatic brain injury is characterized as a hemorrhagic condition compared to cardiac arrest, head trauma should be treated more carefully. Given that coagulopathy adversely affects patients, appropriate blood transfusions are necessary at the initial point of care; abnormalities in electrolytes such as K^+^, P^+^, and Mg^2+^ may also be present. Consequently, frequent electrolyte monitoring and prophylactic correction is necessary because electrolyte abnormalities can cause potentially fatal arrhythmias; it is recommended that K^+^ levels be maintained above 4.0 mEq/L before induction of hypothermia and 3.0–3.5 mEq/L during maintenance (Madden et al., 2017). Furthermore, Mg^++^ is necessary for Na^+^, K^+^, and Ca^++^ to move in and out of cells, and hypomagnesemia causes hypocalcemia and hypokalemia. Therefore, Mg levels must be properly tested and maintained during hypothermia (Polderman et al., 2001). Additionally, hypothermia can increase insulin resistance and cause hyperglycemia (Jo, 2022), necessitating frequent monitoring of blood glucose levels during hypothermia. These complications are to be expected when managing hypothermia and require careful monitoring during the management process.

## 3 Conclusion

In this review, we have delved into clinical studies, with a focus on RCTs and guidelines pertaining to temperature management. The present review does not comprehensively examine the effects of different patient populations and other factors. However, it is possible that variations in temperature management, such as differences in the cooling rate, cooling method, and warming rate, may have varying influences on the effectiveness of temperature management strategies in different target patients. In the future, it may be imperative to examine the impact of the quality of temperature management techniques on the target patients from various perspectives. The quality of management techniques may warrant discussion in the future.
