# Supplementary material for: A review on targeted temperature management for cardiac arrest and traumatic brain injury
Source: Front Neurosci. 2024 Oct 31;18:1397300. doi: 10.3389/fnins.2024.1397300 (PMC11560895; doi:10.3389/fnins.2024.1397300)
Supplement: Supplementary file 1 [file Table_1.DOCX]

**Supplemental Table S1. Effects of Targeted Temperature Management and Increase in Body Temperature on the Central Nervous System**

| **Effect** | **Cardiac Arrest** | **Traumatic brain injury** |
| --- | --- | --- |
| Effects of TTM on the central nervous system | **Differences** | |
|  | Inhibits cell apoptosis associated with ischemia and reperfusion injury | Suppresses fever due to trauma-induced cytokine production  Suppresses secondary brain injury due to increased cerebral vascular permeability associated with fever and reduced exposure of neuronal cells to cytokines |
|  | **Similarities** | |
|  | Reduces BBB damage  Oligodendrocyte and microglial damage is reduced  Suppresses apoptosis in damaged cells by inhibiting the activation of caspase-9, inhibiting the accumulation of free radicals, decreasing glutamate and mitochondrial dysfunction  Decreased CBF leads to decreased cerebral edema, decreased metabolism, decreased ROS, and decreased apoptosis  Decreased metabolism leads to constriction of cerebral blood vessels, decreased intracranial blood volume, and decreased ICP | |
| Effects of hyperthermia on the central nervous system | Increased vascular permeability and exposure to cytokines  Increased vascular permeability and exacerbated brain edema  Increased inflammatory cell infiltration into damaged brain regions  Increased damage and morphology of axons, neurons, glial cells, and vascular endothelium  Increased cerebral metabolism and increased CBF requirements  Impaired autoregulation of CBF results in increased intracranial pressure with increased body temperature | |

TTM: targeted temperature management, BBB: blood brain barrier, CBF: cerebral blood flow, ROS: reactive oxygen species, ICP: intra cranial pressure
